# Bicornuate Uterus With a Rudimentary Horn: Management and Considerations

**DOI:** 10.7759/cureus.74642

**Published:** 2024-11-28

**Authors:** Laila Alhubaishi, Aya Alsalihi, Alya Sharaf, Jinan Khalifa, Abdulla Sharaf

**Affiliations:** 1 Obstetrics and Gynecology, Latifa Hospital, Dubai, ARE; 2 Obstetrics and Gynecology, Hatta Hospital, Dubai, ARE; 3 Medicine, Gulf Medical University, Ajman, ARE

**Keywords:** bicornuate uterus, hysterosalpingorgram, impairment in the fusion of mullerian ducts, preterm labor, recurrent pregnancy loss

## Abstract

A defect in the fusion of Müllerian ducts results in the uterine malformation of the bicornuate uterus. The bicornuate uterus is an uncommon condition, and it is associated with adverse early pregnancy and antenatal events, such as recurrent miscarriages, preterm labor, and delivery.

The bicornuate uterus has two symmetric uterine cavities that are fused caudally and have some degree of communication between the two cavities, usually at the uterine isthmus. This anomaly led to a heart-shaped uterus instead of a pear shape.

Correction of this anatomical anomaly may result in better obstetric outcomes for the patient. We present a case of a female diagnosed to have a bicornuate uterus and a poor obstetric history who underwent complex laparoscopic surgery for correction of this anomaly.

We present a case of a bicornuate uterus with a history of recurrent miscarriages and its management.

## Introduction

Congenital malformations of the uterus can occur during the development of the Müllerian duct; they occur as a result of a defect in combination with canalization and resorption of the septum.

A defect in the fusion of Müllerian ducts results in the uterine malformation of the bicornuate uterus. The bicornuate uterus is an uncommon condition, and it is associated with adverse early pregnancy and antenatal events, such as recurrent miscarriages, preterm labor, and delivery.

The bicornuate uterus has two symmetric uterine cavities that are fused caudally and have some degree of communication between the two cavities, usually at the uterine isthmus. This anomaly leads to a heart-shaped uterus instead of a pear shape [1].

We present a case of a bicornuate uterus with a history of recurrent miscarriages and its management.

## Case presentation

This case concerns a 42-year-old female patient, married for 18 years.. She was referred to our hospital as a case of uterine anomaly and secondary infertility.

She was previously diagnosed with a bicornuate uterus with a rudimentary horn, a single kidney, and recurrent miscarriages.

Her past obstetric history was significant for seven pregnancies, but no living issue. She had five first-trimester miscarriages, one ectopic pregnancy for which a salpingectomy was done, and one extreme preterm delivery at 26 weeks for a twin pregnancy that was conceived via in vitro fertilization (IVF).

She has been screened for thrombophilia and karyotyping, which were both normal. As part of her work-up, an MRI and hysterosalpingogram were done.

The MRI showed a unicornuate uterus with two horns and communicating cavities (Müllerian duct anomaly type 2a communicating). Both uteri show multiple variable-sized uterine leiomyomata of variable sizes and locations as described. The left horn contains two subendometrial cysts probably representing adenomyosis. The hysterosalpingogram was inconclusive.

The patient was counseled for diagnostic hysteroscopy with laparoscopic subtotal hysterectomy for removal of the rudimentary horn and adenomyosis resection; all risks were explained, and informed consent was obtained.

**Figure 1:**
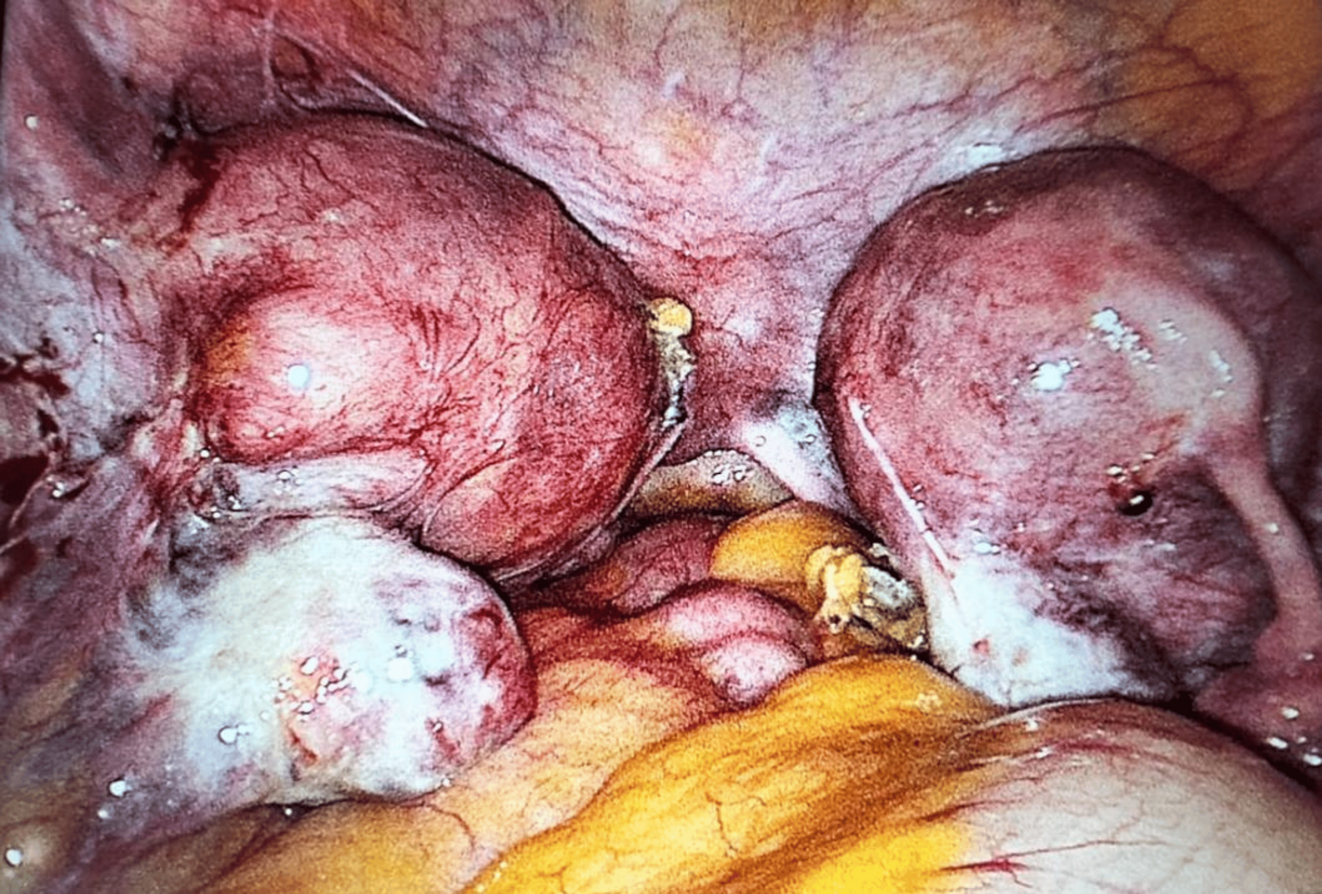
Non-communicating bicornuate uterus, right fallopian tube present.

She underwent the procedure electively. The findings on hysteroscopy and laparoscopy revealed a non-communicating bicornuate uterus, with the left hemi-uterus leading to the cervix, normal ovaries, the presence of the right tube, and absence of the left tube (history of salpingectomy for ectopic) (Figure 1). The upper abdomen was normal, and a single left kidney was noted.

Hysteroscopy showed a normal vagina, a normal single cervix with os deviated toward the left, leading into the left hemiuterus. The cavity had a left os, with the right side being blind. Using a Betocchi scope, the scissors cavity was dilated slightly, and the anti-adhesive uterine gel was inserted into the cavity.

**Figure 2:**
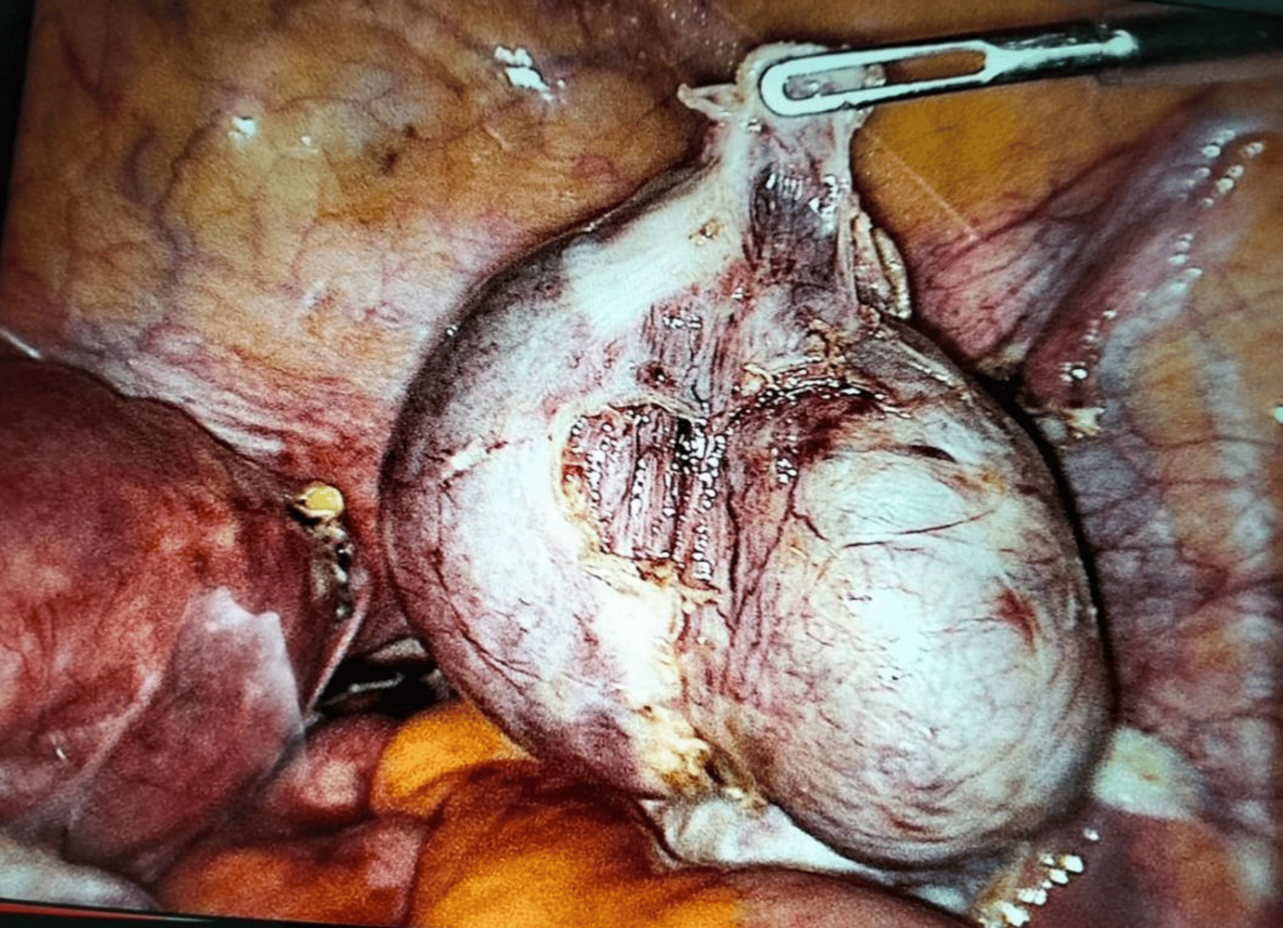
Right tube and fimbria of the rudimentary horn were excised.

Laparoscopy showed a small omental band between the left uterus and the rudimentary horn. On the right side, coagulation and section of the round ligaments were performed using Ligasure. The right ovarian ligament and fallopian tubes were cauterized with Ligasure (Figure 2).

**Figure 3:**
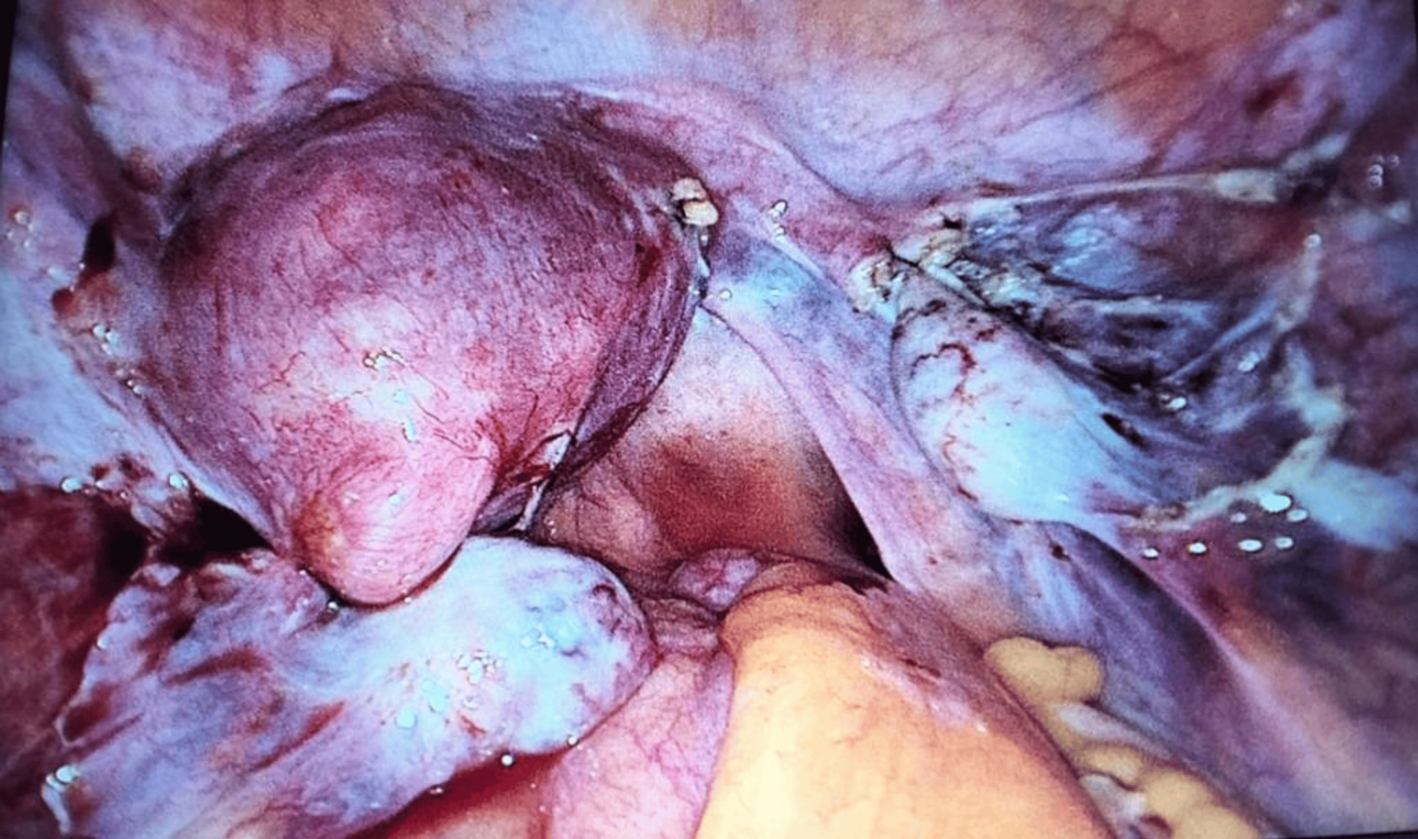
Final look after removal of non-communicating rudimentary horn.

The right hemi-uterus was cut with Ligasure. Morcellation of the right uterus was performed, and the specimen was sent to histopathology. The right ovary was fixed with two sutures, and the infundibulopelvic ligament was long (Figure 3).

The postoperative period was uneventful, and the patient was discharged in good condition.

The histopathology report was as follows: endometrium in the secretory phase, myometrium with adenomyosis and leiomyoma, and the right fallopian tube showing a complete transverse section of a normal fallopian tube.

## Discussion

Müllerian duct anomalies are congenital alterations with more prevalence varying from 0.5% to 6.7% in the general population and up to 16.7% in women with recurrent miscarriage [2].

About 10% to 15% of cases account for anatomic abnormalities of recurrent pregnancy loss (RPL) and are mostly considered to cause miscarriage by interrupting the vasculature of the endometrium, resulting in abnormal and inadequate placentation [3].

A bicornuate uterus is an independent risk factor for cervical os insufficiency as seen in our case with her history of extreme preterm delivery [4].

Nowadays, human chorionic gonadotropin (HCG) is considered one of the diagnostic methods for uterine anomalies due to its low cost and noninvasiveness.

MRI is the modality of choice for evaluating Müllerian duct anomalies of the uterus [5]. However, in our case, it showed the anomaly as unicornuate.

Retrospective studies provide evidence that states the overall rate of miscarriage declines in patients with surgical management of certain congenital and acquired uterine defects. Given the lack of randomized clinical trials, the agreement is that surgical correction should be considered in patients with RPL [6].

Transabdominal metroplasty is considered a successful procedure for a bicornuate uterus, with a prospective study showing an increase in the fetal survival rate from 0% before surgery to 80% after the operation without any intraoperative, postoperative, or intrapartum complications [7].

There is no recommended minimally invasive surgery (MIS) method for a bicornuate uterus [8].

Our case shows a minimally invasive approach to the management of a bicornuate uterus with no intraoperative or postoperative complications; however, the future obstetric outcome still remains to be seen.

## Conclusions

MIS should be considered in the treatment of patients with a history of RPL and uterine anomalies amenable to surgery, as it is likely to improve the obstetric outcome in these patients. More well-designed prospective observational studies are needed so that management guidelines may be drawn for uniform treatment.
